# Evaluation des performances du cytomètre « MUSE AUTO CD4/CD4% » vs « GUAVA AUTO CD4/CD4% » pour la mesure du taux de lymphocytes CD4 chez des patients infectés par le VIH au Cameroun

**DOI:** 10.11604/pamj.2019.32.2.16990

**Published:** 2019-01-03

**Authors:** Ginette Claude Mireille Kalla, Esther Voundi Voundi, Florence Mimo Tanghu, Christiane Medi Sike, Michel Noubom, Joseph Mendimi Nkodo, Justin Amougou, Alexis Ndjolo, Francois-Xavier Mbopi-Keou

**Affiliations:** 1 University of Yaounde I, Faculty of Medicine and Biomedical Sciences, Yaounde, Cameroon; 2 Université des Montagnes, Bangangte, Cameroon; 3 Université de Dschang, Dschang, Cameroon; 4 The Institute for the Development of Africa (The-IDA), Yaounde, Cameroon; 5 Hôpital de District de la Cité Verte, Yaoundé, Cameroon; 6 Chantal Biya International Research Center for HIV/AIDS, Yaounde, Cameroon; 7 UNAIDS Scientific and Technical Advisory Committee (STAC)

**Keywords:** performance, MUSE^®^ Cytometer, GUAVA^®^ Cytometer, CD4, HIV, sub-Saharan Africa, Performance, Cytomètre MUSE^®^, Cytomètre GUAVA^®^, CD4, VIH, Afrique Sub-saharienne

## Abstract

**Introduction:**

Nous avons évalué les performances du cytomètre MUSE^®^, par rapport au cytomètre de référence GUAVA^®^.

**Méthodes:**

Une étude expérimentale a été réalisée sur des échantillons de patients séropositifs au VIH. Les échantillons de sang veineux recueillis dans un tube K3 EDTA, ont été analysés dans un délai de 24-48h par les cytomètres MUSE^®^ et GUAVA^®^, au Centre International de Diagnostic médical de Yaoundé.

**Résultats:**

Au total, 227 échantillons ont été analysés. Il y avait une forte corrélation intraclasse (p<0,0001) entre les cytomètres MUSE^®^ et GUAVA^®^ avec un coefficient de 0,998 (95% IC: 0,998-0,999) pour les valeurs absolues et de 0,992 (95% IC: 0,989-0,994) pour les pourcentages. Une forte corrélation linéaire positive (p=0,000) a été retrouvée entre les cytomètres MUSE^®^ et GUAVA^®^ avec des pentes r2=0,98 (95% IC=0,97-0,99) pour les valeurs absolues et r2= 0,98 (95% IC= 0,96-1,00) pour les pourcentages. Les biais étaient de -4,80 cells/µl (-101,31-91,71) pour les valeurs absolues et de -0,89% (IC: -6,08-4,3) pour les pourcentages. Le pourcentage des points de données en dehors des limites d'accord était de 12/227 (5,29%) et de 10/227 (4,41%) respectivement pour les valeurs absolues et les pourcentages. Le coefficient Kappa de Cohen était de 0,92 et l'aire sous la courbe de 0,9975 (IC 95%: 0,99-1).

**Conclusion:**

Le cytomètre MUSE^®^AUTO CD4/CD4% est un appareil performant car ses résultats concordent avec ceux donnés par le cytomètre de référence, et peut de fait permettre le suivi des patients infectés par le VIH notamment dans les pays en développement.

## Abstract (en)

**Introduction:**

This study aimed to evaluate the performances of the MUSE^®^ flow cytometer compared with the reference GUAVA^®^ flow cytometer.

**Methods:**

We conducted an experimental study on HIV-infected patient samples. Venous blood samples, collected in a K3 EDTA tube, were analyzed within 24-48 hours by MUSE^®^ and GUAVA^®^ cytometers at the International Center for medical diagnosis (Centre International de Diagnostic médical) in Yaoundé.

**Results:**

In total, 227 samples were analyzed. There was a strong intraclass correlation (p<0.0001) between MUSE^®^ and GUAVA^®^ cytometers with a correlation coefficient 0.998 (95% CI: 0,998-0,999) for the absolute values and 0,992 (95% CI: 0,989-0,994) for the percentages. A strong positive linear correlation (p=0.0001) was found between MUSE^®^ and GUAVA^®^ cytometers with linear regression slope r2 = 0.98 (95% CI=0,97-0,99) for the absolute values and r2= 0.98 (95% CI= 0.96-1,00) for the percentages. The biases were -4,80 cells/µl (-101.31-91.71) for the absolute values and -0.89% (IC: -6,08-4.3) for the percentages. The percentage of data points outside the limits of agreement was 12/227 (5.29%) and 10/227 (4.41%) respectively for the absolute values and percentages. Cohen's kappa coefficient was 0.92 and the area under the curve was 0,9975 (CI 95%: 0.99-1).

**Conclusion:**

MUSE^®^AUTO CD4/CD4% cytometer is a powerful instrument because its results are consistent with those obtained by the reference cytometer. It can enable tracking of patients infected with HIV, in particular in the developing countries

## Introduction

Les couts associés au traitement du Syndrome de l'immunodéficience acquise (SIDA), affection meurtrière [1] et des infections opportunistes ne cessent d'augmenter et l'affectation des ressources, déjà limitées, au VIH/SIDA détourne l'attention d'autres préoccupations de santé [2]. Il en ressort la nécessité d'un diagnostic, d'une prise en charge et d'un suivi des patients moins onéreux et tout aussi efficaces [3]. L'Organisation Mondiale de la Santé (OMS) recommande la mesure de la charge virale comme la méthode de suivi privilégiée pour déterminer et confirmer l'échec thérapeutique [4]. Néanmoins, la numération des CD4 reste d'actualité. Si le test de la charge virale n'est pas disponible en routine, le diagnostic d'échec thérapeutique doit reposer sur un suivi de la numération des CD4 et un suivi clinique [4]. Le nombre de CD4 est aussi un facteur prédictif de l'état de l'infection et du risque immédiat de décès [4]. Tous les patients entrant pour la première fois ou à nouveau dans la filière de soins doivent se voir proposer une numération des CD4 au moment de démarrer le traitement et lorsqu'il est cliniquement indiqué de le faire pour les patients instables ou présentant une infection à VIH à un stade avancé. Il est vivement recommandé de proposer aux patients présentant une forme clinique d'infection à VIH avancée (numération des CD4 inférieure à 200 cellules/mm3) un ensemble de soins tel que décrit dans les Lignes directrices pour la prise en charge de l'infection à VIH à un stade avancé et le démarrage rapide d'un traitement antirétroviral de l'OMS [5]. La numération des CD4 se fait grâce à de nombreuses méthodes dont la référence est la technique automatisée par la cytométrie de flux. L'implémentation de cette méthode reste entravée dans les pays à ressources limitées comme le Cameroun, d'où l'importance d'étudier des alternatives adaptables à notre contexte [6, 7] telles que le cytomètre « MUSE^®^ AUTO CD4/CD4% ». Il apparait nécessaire d'évaluer cet appareil moins onéreux, d'accès facile par rapport à d'autres appareils de référence tels que le « GUAVA^®^ AUTO CD4/CD4% ».

## Méthodes

Une étude quasi-expérimentale a été réalisée de janvier à juin 2015. La population d'étude était constituée d'échantillons de patients séropositifs au VIH recrutés de manière consécutive, après obtention du consentement éclairé et de l'assentiment éclairé des tuteurs, au Centre International de Référence Chantal BIYA et à l'Hôpital de District de la Cité Verte dans la ville de Yaoundé. Les échantillons de sang veineux recueillis ont été analysés dans un délai de 24-48 heures par les cytomètres « MUSE^®^ AUTO CD4/CD4% » et « GUAVA^®^ AUTO CD4/CD4% » au Centre International de Diagnostic Médical de Yaoundé. Les variables d'étude étaient les valeurs absolues et les pourcentages des lymphocytes T CD4. Les données ont été analysées grâce aux logiciels IBM-SPSS vs 21 et STATA vs 14 avec pour valeur statistiquement significative p < 0,05. L'évaluation de la performance s'est faite quantitativement et qualitativement. Quantitativement, les corrélations interclasses ont été évaluées grâce au coefficient de corrélation intra classe, la corrélation linéaire grâce à la régression linéaire et la concordance quantitative par la méthode de Bland Altman [8]. Qualitativement, la concordance qualitative a été évaluée à l'aide du coefficient Kappa de Cohen [9] et la capacité prédictive du « MUSE^®^ AUTO CD4/CD4% » à l'aide de la courbe ROC et de l'aire sous la courbe [10]. Concernant la concordance qualitative, les valeurs absolues des CD4 ont été classées en 3 catégories: < 200/mm^3^, entre 200-350/mm^3^ et > 350/mm^3^ et pour la capacité prédictive, les valeurs du « GUAVA^®^ AUTO CD4/CD4% » ont été considérées comme qualitatives et celles du « MUSE^®^ AUTO CD4/CD4% » quantitatives.

## Résultats

Au total, 227 patients ont été inclus dans l'étude parmi lesquels 133 (58,6%) de sexe féminin et 94 (41,4%) de sexe masculin. L'âge médian était de 38 ans avec un espace interquartile allant de 29 à 51 ans.

**Figure 1:**
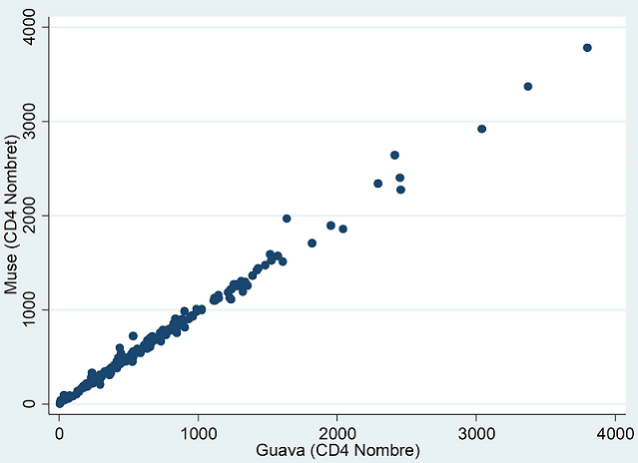
Corrélation entre MUSE^®^ et GUAVA^®^ pour les valeurs absolues de CD4

**Figure 2:**
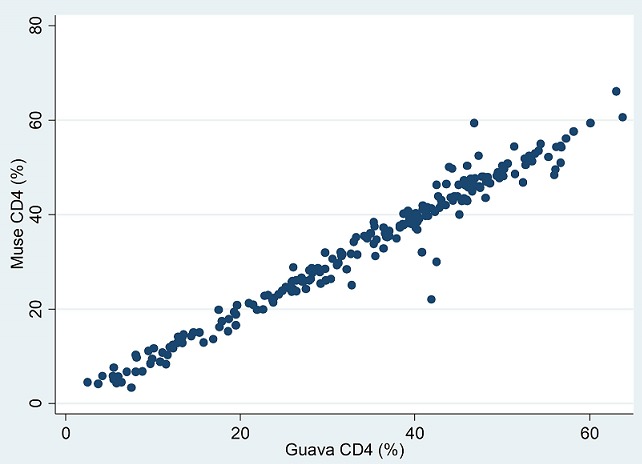
Corrélation entre MUSE^®^ et GUAVA^®^ pour les valeurs en pourcentage de CD4

**Corrélation:** Les coefficients de corrélation intra classe ont été chiffrés à 0,998 (95%IC: 0,998-0,999) pour les valeurs absolues et à 0,992 (95%IC: 0,989-0,994) pour les valeurs en pourcentage (p<0,0001). De plus, la régression linéaire montre des pentes chiffrées (r2) à 0,98 (95%IC: 0,97-0,99; p=0,000) pour les valeurs absolues (Figure 1) et à 0,98 (95%IC: 0,96-1,00; p=0,000) pour les pourcentages (Figure 2)

**Figure 3:**
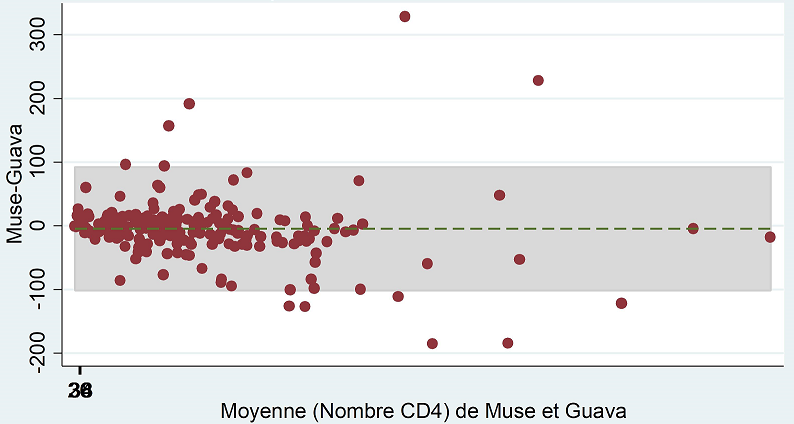
Concordance entre MUSE^®^ et GUAVA^®^ pour les valeurs absolues de CD4

**Figure 4:**
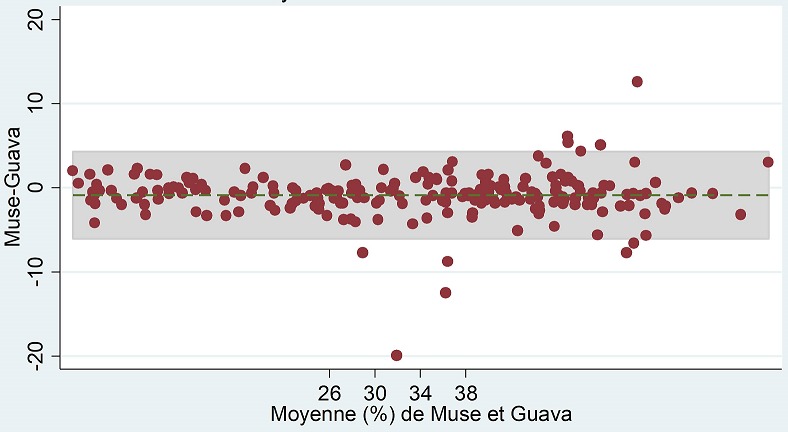
Concordance entre le MUSE et le GUAVA pour les valeurs en pourcentage de CD4

**Analyse de Bland Altman:** Les valeurs du biais (limites d'accord) étaient de -4,80 cellules/µL (-101,31- 91,71) pour les valeurs absolues (Figure 3) et de -0,89% (-6,08- 4,30) pour les pourcentages (Figure 4). De plus, les taux des points de données en dehors des limites d'accord de l'étude étaient de 12/227 (5,3%) pour les valeurs absolues et de 10/227 (4,4%) pour les pourcentages.

**Concordance qualitative:** Sur 33 échantillons avec CD4< 200/mm^3^ retrouvés par le « GUAVA^®^ AUTO CD4/CD4%», le « MUSE^®^ AUTO CD4/CD4% » en avait diagnostiqué 31 avec un taux de CD4 < 200/mm^3^ soit 94%. Sur 33 échantillons avec CD4 de 200-350/mm3 retrouvés par le « GUAVA AUTO CD4/CD4%», le « MUSE^®^ AUTO CD4/CD4% » en avait diagnostiqué 31 avec un taux de CD4 entre 200-350/mm^3^ soit 94%. Sur 161 échantillons avec CD4 >350/mm^3^ retrouvés par le « GUAVA^®^ AUTO CD4/CD4%», le « MUSE^®^ AUTO CD4/CD4% » en avait diagnostiqué 157 avec un taux de CD4 >350/mm3 soit 97,5% (Tableau 1). Cette analyse a révélé un coefficient Kappa de Cohen de 0,92.

**Tableau 1:** Concordance qualitative entre les cytomètres SE^®^AUTOCD4/CD4% et GUAVA^®^AUTOCD4/CD4%

	MUSE^®^			
**Taux de CD4**	**<200 n(%)**	**200-350 n(%)**	**>350 n(%)**	**Total n(%)**
<200	**31(94)**	2(6)	0(0)	33(14,5)
200-350	2(6)	**31 (94)**	0(0)	33(14,5)
GUAVA^®^ >350	0(0)	4(2,5)	**157(97,5)**	161(71)
Total	33(14,5)	37(16,2)	157(69,1)	227(100)

**Figure 5:**
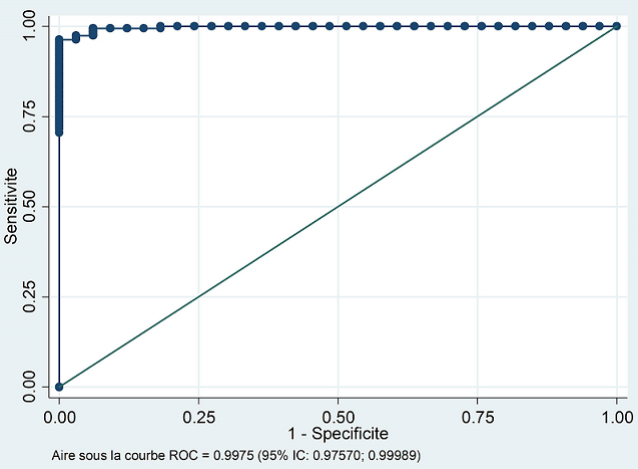
Courbe ROC pour la sensibilité et la spécificité du cytomètre MUSE^®^ par rapport au cytomètre GUAVA^®^

**Capacité prédictive avec courbe ROC et Aire sous la courbe:** La courbe caractéristique ROC (Figure 5) a montré de fortes sensibilités et spécificités pour la majorité des valeurs du « MUSE^®^ AUTO CD4/CD4% » avec une aire sous la courbe de 0,998 (95%IC: 0,978-0,999).

## Discussion

Dans la présente étude, l'objectif était d'évaluer les performances du cytomètre en flux « MUSE^®^ AUTO CD4/CD4% » en prenant pour référence le cytomètre « GUAVA^®^ AUTO CD4/CD4%» pour la mesure des lymphocytes T CD4 exprimés en valeurs absolues et en pourcentages. L'évaluation a pris en compte la corrélation, la concordance et la capacité prédictive entre les deux cytomètres. De fortes corrélations ont été retrouvées entre le « MUSE^®^ AUTO CD4/CD4% » et le « GUAVA^®^ AUTO CD4/CD4%». La corrélation était maintenue sur presque toute la gamme dynamique des valeurs avec un coefficient de 0,998 et de 0,992 pour les valeurs absolues et pour les pourcentages respectivement. Ces résultats concordaient avec ceux retrouvés dans une étude faite par Mossoro-Kpinde et collaborateurs, en République Centrafricaine en 2016, sur une évaluation du cytomètre « MUSE^®^ AUTO CD4/CD4%» par rapport au FASCOUNT^®^ [10]. Cette étude a retrouvé des coefficients de corrélation de 0,99 et 0,98 pour les valeurs absolues et pour les pourcentages des lymphocytes CD4 respectivement. De même dans l'étude de Hartig et collaborateurs, en 2014 aux Etats-Unis, qui comparait le « MUSE^®^ AUTO CD4/CD4% » et le « GUAVA^®^ AUTO CD4/CD4%», une forte corrélation avec des coefficients de corrélation de 0,99 et 0,99 pour les valeurs absolues et pour les pourcentages des lymphocytes CD4 respectivement; ce qui corroborait nos résultats.

L'étude de la concordance quantitative entre les appareils a montré que les valeurs du biais (limites d'accord) étaient de -4,80 cellules/µL (-101,31-91,71) pour les valeurs absolues et de -0,89% (-6,08- 4,30) pour les pourcentages. Ces résultats étaient similaires ceux retrouvés dans l'étude de Mossoro-Kpinde CD et collaborateurs qui retrouvait des résultats de biais de -5,91 cellules/µL (-77,50-202,40) pour les valeurs absolues et de +1,69% (1,29-2,09) pour les pourcentages de lymphocytes CD4 [11]. Selon la courbe ROC, l'aire sous la courbe était de 0,998, ce qui sous-tend de fortes sensibilités et spécificités des résultats fournis par le « MUSE^®^ AUTO CD4/CD4% ». Ce résultat concordait avec celui retrouvé dans l'étude de Hartig et collaborateurs [12].

## Conclusion

Au vu de nos résultats, il en ressort une forte corrélation, une concordance excellente montrant l'interchangeabilité possible et une capacité prédictive presque parfaite du cytomètre « MUSE AUTO CD4/CD4% » par rapport au « GUAVA AUTO CD4/CD4%». Aussi, le cytomètre « MUSE AUTO CD4/CD4% » pourrait faciliter de fait, le suivi des patients infectés par le VIH dans les pays en développement à l'instar de ceux d'Afrique sub-saharienne.

### Etat des connaissances actuelles sur le sujet

- Le cytomètre « MUSE AUTO CD4/CD4% » est performant dans d'autres pays où il a été évalué.

### Contribution de notre étude à la connaissance

- Le cytomètre « MUSE AUTO CD4/CD4% » est tout aussi performant que le « GUAVA AUTO CD4/CD4%»;
- Le cytomètre « MUSE AUTO CD4/CD4% » peut être utilisé dans les pays en développement à l'instar de ceux d'Afrique sub-saharienne comme alternative plus accessible.

## Conflits d’intérêts

Les auteurs ne déclarent aucun conflit d'intérêts.

## Remerciements

Nos remerciements s'adressent à tous nos patients et à leurs tuteurs pour leur participation à l'étude, à Pierrette Omgba Bassega et Samuel Sosso pour le recrutement des patients. Des remerciements particuliers à Merck (Germany) pour la fourniture des reactifs et cytomètres MUSE^®^ et GUAVA^®^, notamment à Kamala Tyagarajan, Julie Clor, Philippe Durbiano et Jim Mulry. Enfin, nous remercions Wilfried Mbacham, Kamala Tyagarajan et Fru F Angwafor III pour la lecture critique du manuscript.
